# Retraction: Association of FcεRIβ polymorphisms with risk of asthma and allergic rhinitis: Evidence based on 29 case-control studies

**DOI:** 10.1042/BSR-2018-0177_RET

**Published:** 2022-09-12

**Keywords:** allergic diseases, FcεRIβ, genetic model, Meta-analysis, polymorphism

This article is being retracted from *Bioscience Reports* at the request of the first author due to data having been lost and being unable to provide answers for errors identified in the paper. This Retraction follows an Expression of Concern relating to this article previously published by Portland Press, along with a correspondence letter commenting on the article (which can be seen at https://doi.org/10.1042/BSR20193424). The corresponding author has been contacted but has not responded to the Journal’s queries or the concerns raised. The first author is unable to correct the article and wishes to retract the article. The Editor-in-Chief and Editorial Board agree with the Retraction.

